# HIV Incidence Remains High in KwaZulu-Natal, South Africa: Evidence from Three Districts

**DOI:** 10.1371/journal.pone.0035278

**Published:** 2012-04-19

**Authors:** Annaléne Nel, Zonke Mabude, Jenni Smit, Philip Kotze, Derek Arbuckle, Jian Wu, Neliëtte van Niekerk, Janneke van de Wijgert

**Affiliations:** 1 International Partnership for Microbicides, Silver Spring, Maryland United States of America; 2 MatCH, Department of Obstetrics and Gynecology, University of the Witwatersrand Durban, KwaZulu-Natal, South Africa; 3 Qhakaza Mbokodo Research Clinic, Ladysmith, KwaZulu-Natal, South Africa; 4 PHIVA Project, Pinetown, KwaZulu-Natal, South Africa; 5 JW Consulting, Hurstville Grove, New South Wales, Australia; 6 International Partnership for Microbicides, Paarl, Western Cape, South Africa; 7 Academic Medical Center of the University of Amsterdam and Amsterdam Institute for Global Health and Development, Amsterdam, The Netherlands; 8 International Partnership for Microbicides, Paarl, Western Cape, South Africa; University of Washington, United States of America

## Abstract

**Background:**

HIV prevalence and incidence among sexually active women in peri-urban areas of Ladysmith, Edendale, and Pinetown, KwaZulu-Natal, South Africa, were assessed between October 2007 and February 2010 in preparation for vaginal microbicide trials.

**Methodology/Principal Findings:**

Sexually active women 18–35 years, not known to be HIV-positive or pregnant were tested cross-sectionally to determine HIV and pregnancy prevalence (798 in Ladysmith, 1,084 in Edendale, and 891 in Pinetown). Out of these, approximately 300 confirmed non-pregnant, HIV-negative women were subsequently enrolled at each clinical research center (CRC) in a 12-month cohort study with quarterly study visits. Women in the cohort studies were required to use a condom plus a hormonal contraceptive method. HIV prevalence rates in the baseline cross-sectional surveys were high: 42% in Ladysmith, 46% in Edendale and 41% in Pinetown. Around 90% of study participants at each CRC reported one sex partner in the last 3 months, but only 14–30% stated that they were sure that none of their sex partners were HIV-positive. HIV incidence rates based on seroconversions over 12 months were 14.8/100 person-years (PY) (95% CI 9.7, 19.8) in Ladysmith, 6.3/100 PY (95% CI 3.2, 9.4) in Edendale, and 7.2/100 PY (95% CI 3.7, 10.7) in Pinetown. The 12-month pregnancy incidence rates (in the context of high reported contraceptive use) were: 5.7/100 PY (95% CI 2.6, 8.7) in Ladysmith, 3.1/100 PY (95% CI 0.9, 5.2) in Edendale and 6.3/100 PY (95% CI 3.0, 9.6) in Pinetown.

**Conclusions/Significance:**

HIV prevalence and incidence remain high in peri-urban areas of KwaZulu-Natal.

## Introduction

The South African province of KwaZulu-Natal is experiencing one of the worst HIV epidemics worldwide. The epidemic has been described as hyperendemic, generalized and mature, with HIV prevalence rates in the general population of over 15% [1]–[5]. Data from the Department of Health antenatal surveys and the Human Sciences Research Council (HSRC) cross-sectional population-based household surveys have shown a stabilization of prevalence rates since 2005 [3]–[5]. HIV prevalence is expected to increase in the context of a mature epidemic with increasing access to antiretroviral therapy because people living with HIV will survive longer [6], [7]. Therefore, HIV incidence data are increasingly important because only data on new HIV infections will provide insight into ongoing transmission dynamics [6], [7].

KwaZulu-Natal is divided into 11 districts. The HIV epidemics in two of these districts have been extensively studied: the urban district of eThekwini (Durban Metropolitan Area and surrounding area) and the rural district of uMkhanyakude (where the Africa Centre of the University of KwaZulu-Natal is located) [8]–[11]. Recent studies in these districts found HIV incidence rates of 6.4/100 person-years (PY) among urban women and 6.5/100 PY among rural women aged 14–30 years [8] and 3.6/100 PY among rural women aged 15–55 [11]. Our research was conducted in eThekwini district (in the small town of Pinetown, about 16 km west of Durban) and in two under-researched districts of KwaZulu-Natal: Ladysmith, the capital city of the uThukela district, and Edendale (near Pietermaritzburg) in the uMgungundlovu district. All three study areas can be characterized as peri-urban. According to the 2009 and 2010 antenatal surveys, the HIV prevalence rates in pregnant women in eThekwini, uThukela, and uMgungundlovu districts were 41.4/41.1%, 46.4/36.7%, and 40.9/42.3%, respectively [3], [4].

We conducted HIV prevalence and incidence studies in sexually active women in the above-mentioned peri-urban areas of KwaZulu-Natal to better understand how many and where new HIV infections are occurring and to assess the feasibility of undertaking vaginal microbicide trials for HIV prevention in these populations.

## Methods

### Study design and populations

In preparation for future vaginal microbicide trials for HIV prevention in KwaZulu-Natal, cross-sectional studies (targeting 800–1,000 women each) were conducted at three clinical research centers (CRC) to determine HIV prevalence and to identify HIV-negative, non-pregnant women for enrollment in subsequent cohort studies (targeting 300 women each). The main aim of the cohort studies was to determine HIV incidence in seroconversions per 100 PY. The CRCs were located in Ladysmith, Edendale, and Pinetown. Each CRC established a Community Advisory Group (CAG) to provide community input in study procedures and to assist the researchers with community education and mobilization. CRC staff, with the assistance of CAG members, organized meetings in public spaces (at public meetings, in shopping centers and in waiting areas of clinics), where the study was presented. Women who expressed an interest in study participation were then invited to visit the CRC for screening and possible enrollment. In addition, door-to-door or family visits were conducted by study staff. While the recruitment strategies were CRC-specific, the same study procedures were followed at each CRC from the moment women were screened for study participation.

Women were eligible for the cross-sectional studies if they were 18–35 years, not HIV-positive or pregnant by self-report, not breastfeeding, and sexually active (defined as at least one penetrative vaginal coital act per month for the previous three months). Women who tested HIV- and pregnancy-negative in the cross-sectional studies, still met the entry criteria described above, and met additional entry criteria for the cohort studies, were subsequently offered enrollment into the cohort studies. These additional entry criteria included using a condom plus a hormonal (oral or injectable) contraceptive method [12], not injecting non-therapeutic drugs, not participating in other studies, not suffering from specified chronic diseases or allergies, refraining from anal sex and planning to stay in the study area for the duration of the study. Follow-up visits were scheduled at 3, 6, 9 and 12 months.

At all study visits, women were interviewed regarding demographics, sexual behavior, vaginal hygiene practices, and medical history; and received HIV risk reduction and contraceptive counseling, condoms, and syndromic management of sexually transmitted infections (STI) free of charge [13]. Confirmed HIV-positive women were referred for HIV care, and pregnant women were referred for antenatal care. HIV-positive and pregnant women enrolled in the cohort studies could continue study participation if so desired. The study was approved by two ethical review committees in South Africa: the University of Witwatersrand Human Research Ethics Committee and Pharma-Ethics. Formal support for the study was also obtained from provincial, district, hospital and clinical authorities, and from local community leaders. Written informed consent was obtained from all study participants.

### Laboratory testing

An HIV testing algorithm was used to determine the presence of prevalent and incident HIV infections. Women were first tested by OraQuick ADVANCE Rapid HIV-1/2 Antibody Test using oral swabs (OraSure Technologies Inc., Bethlehem, PA, USA) or by Uni-Gold Recombigen HIV test using blood (Trinity Biotech, Bray, Wicklow, Ireland). When this first HIV test was positive, blood samples were tested by Determine HIV-1/2 rapid test (Inverness Medical Professional Diagnostics, Princeton, NJ, USA), and by enzyme-linked immunosorbant assay (ELISA) if a tiebreaker was needed. Blood samples from women who were confirmed HIV-positive were also tested by BED assay (Calypte Biomedical Corporation, Portland, OR, USA) according to the manufacturer's instructions. A specimen with a final normalized optical density value of less than or equal to 0.8 was considered to be from a participant who was infected less than 155 days before [14]. Urine samples from each participant were tested for pregnancy using an hCG pregnancy test.

### Data Analysis and Statistics

Case report forms were processed using the DataFax data management system (Clinical DataFax Systems Inc., Hamilton, Ontario, Canada) and analyzed using SAS version 9.2 (SAS Institute, Cary, NC, USA). Descriptive statistics were used to summarize baseline demographic, behavioral and clinical characteristics. Categorical variables were expressed as percentages, and continuous data as medians with inter-quartile ranges.

Incidence rates in the cohort studies were calculated based on a Poisson distribution with PY at risk in the denominator. A person's time at risk began at the enrollment visit and ended at the last study visit attended (usually the Month 12 visit) or when HIV infection or pregnancy occurred. HIV infection and pregnancy were assumed to have occurred at the mid-point between the last available negative test and first positive test. A woman who reached an HIV endpoint was no longer considered at risk for HIV but was still considered at risk for pregnancy, and vice versa. HIV incidence rates and 95% confidence intervals based on BED results in the cross-sectional studies were calculated using the formula, and accompanying spreadsheet, provided by McWalter and Welte [15], [16]. Inputs in the formula include the total number of HIV-positive and HIV-negative individuals in the sample, the number of HIV-positive individuals who also tested positive on the BED assay, the BED window period, and an estimated BED false-recent rate (FRR). A recent study in KwaZulu-Natal found a local FRR of 1.7% [17] and a study in Zimbabwe a window period of 187 days (instead of the 155 days that are specified in the package insert) [18]. Two BED adjustments were therefore made: one using a window period of 155 days and a FRR of 1.7%, and another one using a window period of 187 days and a false recent rate of 1.7%. Incidence estimates are expressed as an incidence rate (number of new HIV infections per 100 PY).

Age-adjusted and multivariable logistic regression models were used to assess predictors of prevalent HIV infection and pregnancy, with p-values from the Wilcoxon-Mann-Whitney test for continuous variables and the Chi-square and Fisher's exact tests for categorical variables. Age-adjusted Cox proportional hazards regression models were used to assess predictors of HIV seroconversion and incident pregnancy.

## Results

### Disposition

Women were enrolled in the cross-sectional studies between 2007 and 2009 as follows: 798 women in Ladysmith, 1,084 women in Edendale, and 891 women in Pinetown. The Ladysmith and Edendale CRCs subsequently enrolled 300 women in their cohort studies and the Pinetown CRC 297 women, accumulating 223, 254, and 223 PY respectively. In Ladysmith, 129 of 300 (43%) participants completed all scheduled visits; 53 women withdrew early from the cohort study, 32 were lost to follow-up, and none died. In Edendale, 210 of 300 (70%) participants completed all scheduled visits; 6 women withdrew early from the cohort study, 24 were lost to follow-up, and none died. In Pinetown, 167 of 297 (56%) participants completed all scheduled visits; 5 women withdrew early from the cohort study, 74 were lost to follow-up, and none died.

### Demographic and behavioral characteristics

In the cross-sectional studies, the median age of study participants was 23 or 24 years (Table 1). Almost all participants were black African, and more than 80% at each CRC was single, had only one sexual partner in the last 3 months, and had at least some high school education. About half of the participants (47–62%) had used a condom during their last sex act, while only 14–30% was sure that they did not currently have a sex partner who was HIV-positive. Anal sex was rarely reported at each CRC (<3%), but oral sex was more common (14–16%). Women in Pinetown were more likely to report cleansing or drying the vagina before or after sex (8% and 4%, respectively) than women in Ladysmith and Edendale. Less than 4% of all women reported a genital symptom. At each CRC, demographic and sexual behavior characteristics of cohort study participants at enrollment were similar to cross-sectional participants. However, fewer women in the cohort than in cross-sectional studies felt that they were at high risk for HIV (25% vs. 41% in Ladysmith, 22% vs. 40% in Edendale, and 31% vs. 47% in Pinetown). Furthermore, women enrolled in the cohort in Edendale reported more condom use during the last sex act than those enrolled in the cross-sectional study (69% vs. 53%).

**Table 1:** Characteristics of Cross-sectional Study Participants.

	Ladysmith	Edendale	Pinetown
Characteristic n (%)	N = 798	N = 1,084	N = 891
Age in years (median; range)	24 (18–35)	24 (18–35)	23 (18–35)
Race: Black African	792 (99.2)	1,081 (99.7)	890 (99.9)
Marital status			
Single	723 (90.6)	988 (91.1)	799 (89.7)
Married/or living together	75 (9.4)	90 (8.3)	91 (10.2)
Separated/divorced/widowed	0	6 (0.6)	1 (0.1)
Education			
No school	2 (0.3)	5 (0.5)	3 (0.3)
Some/completed primary school	26 (3.3)	40 (3.6)	81 (9.1)
Some/completed high school	651 (81.6)	1,002 (92.5)	748 (84.3)
Some/completed tertiary school	119 (14.9)	36 (3.3)	55 (6.2)
Source of income^1^			
Formal/informal work	193 (24.2)	56 (5.2)	123 (13.8)
Government grants	321 (40.2)	639 (59.1)	509 (57.2)
Husband/partner	95 (11.9)	25 (2.3)	78 (8.8)
Other	471 (59.0)	397 (36.7)	234 (26.3)
Average monthly income			
0-R500	542 (67.9)	1034 (95.7)	772 (86.6)
>R500	256 (32.1)	47 (4.3)	119 (13.4)
Male sex partners in last 3 months			
1	718 (90.0)	998 (92.1)	780 (87.5)
2 or more	80 (10.0)	86 (7.9)	111 (12.5)
Male sex partners in last 7 days			
0	152 (19.9)	215 (19.9)	135 (15.2)
1	608 (79.5)	859 (79.3)	750 (84.4)
2 or more	5 (0.7)	9 (0.8)	4 (0.4)
Condom used during last sex act	374 (46.9)	577 (53.2)	550 (62.0)
Condom use in last 7 day^2^			
Always	228 (35.5)	404 (42.2)	322 (42.2)
Inconsistent	197 (30.7)	137 (14.3)	182 (23.9)
Never	217 (33.8)	416 (43.5)	259 (33.9)
Any chance that any current sex partner is HIV-positive			
Yes	210 (26.3)	178 (16.7)	182 (20.5)
No	235 (29.5)	187 (17.5)	125 (14.1)
Don't know	352 (44.2)	702 (65.8)	579 (65.3)
Ever had anal sex	4 (0.5)	28 (2.6)	3 (0.3)
Ever had oral sex	115 (14.4)	169 (15.6)	145 (16.3)
Ever vaginal cleansing before or after sex	8 (1.0)	20 (1.8)	67 (7.6)
Ever vaginal drying before or after sex	1 (0.1)	16 (1.5)	37 (4.1)
Self assessment of HIV risk^3^			
No/low risk	392 (51.4)	449 (42.2)	407 (50.1)
Moderate risk	58 (7.6)	188 (17.7)	20 (2.5)
High risk	312 (40.9)	426 (40.1)	385 (47.4)
Reported genital symptom at baseline^4^	34 (4.3)	15 (1.4)	27 (3.0)

1 Multiple responses allowed.

2 Women who reported any sexual intercourse in the last 7 days only.

3 Women who said ‘don't know’ were excluded.

4 Includes lower abdominal pain, genital discharge, odor, ulcers, sores, itching or swelling, burning pain on urination.

### Condom use dynamics

More than 80% of women at all three CRCs reported that they themselves, or they and their partner together, decided about condom use (data not shown). About one third of women (28% in Ladysmith, 16% in Edendale, and 39% in Pinetown) reported to have refused sex in the last 7 days due to lack of a condom. The most common reasons for using a condom were ‘to protect myself from HIV’ (49% in Ladysmith, 74% in Edendale, and 70% in Pinetown), followed by ‘to prevent pregnancy’ (41% in Ladysmith, 66% in Edendale, and 52% in Pinetown), and ‘to protect myself from STIs’ (29% in Ladysmith, 38% in Edendale, and 58% in Pinetown). Protecting sexual partners from HIV or STIs was less often mentioned in Ladysmith and Edendale, and rarely mentioned in Pinetown (data not shown). The most common reason for not using a condom was partner refusal (40% in Ladysmith, 28% in Edendale, and 33% in Pinetown).

### HIV prevalence

HIV prevalence was higher than 40% at all three CRCs: 42.0% (95% CI 38.5, 45.5) in Ladysmith, 46.1% in Edendale (95% CI 43.1, 49.1), and 41.3% (95% CI 38.0, 44.6) in Pinetown. Factors positively associated with prevalent HIV infection at all three CRCs in age-adjusted and multivariable models were: age, lower educational level, self-assessment of HIV risk as moderate or high (compared to no or low risk), and suspected positive or unknown HIV serostatus of a current sexual partner; no or inconsistent condom use was associated with HIV infection in all age-adjusted models but not in all multivariable models (Tables 2 and 3). Having an income below 500 Rand per month, having more than one sex partner in the last 3 months, and the presence of genital symptoms at baseline were only associated with prevalent HIV in Pinetown (Tables 2 and 3). Being married or living together and oral sex were not associated with prevalent HIV.

**Table 2:** Age-adjusted Determinants of Prevalent HIV Infection in the Cross-Sectional Studies^1^.

Determinant	Ladysmith (N = 798)		Edendale (N = 1,084)		Pinetown (N = 891)	
	% HIV+	Age-adjusted OR (95% CI)	% HIV+	Age-adjusted OR (95% CI)	% HIV+	Age-adjusted OR (95% CI)
Marital status:						
Married/living together	48.0	0.8 (0.5, 1.3)	60.0	1.0 (0.6, 1.5)	53.3	1.2 (0.8, 2.0)
Single, separated or divorced (reference)	41.4		44.8		39.9	
Highest level of education achieved:						
Some/completed primary education	50.0	2.9 (1.2, 7.4)	70.0	9.1 (2.9, 28.9)	54.3	6.2 (2.5, 15.1)
Some/completed high school	45.3	3.0 (1.9, 4.9)	46.2	4.3 (1.7, 10.7)	41.8	3.7 (1.7, 8.1)
Some/completed tertiary education (reference)	21.8		16.7		14.8	
Source of income:						
Formal/informal work (reference)	53.9		48.2		44.2	
Government grants	36.4	0.6 (0.4, 0.9)	52.1	1.2 (0.7, 2.1)	46.8	1.1 (0.7, 1.7)
Husband/Other	39.4	0.9 (0.6, 1.3)	35.9	1.0 (0.5, 1.8)	30.7	0.9 (0.5, 1.4)
Average monthly income^5^						
0-R500 (reference)	40.2		45.8		42.5	
>R500	45.7	0.8 (0.6, 1.1)	51.1	1.1 (0.6, 2.0)	33.3	0.5 (0.3, 0.8)
Number of sex partners in last 3 months						
1 (reference)	40.7		46.3		40.1	
More than 1	53.8	1.9 (1.2, 3.2)	43.0	1.2 (0.7, 1.9)	49.5	1.7 (1.1, 2.6)
Condom use in last 7 days						
Always (reference)	27.2		32.4		32.5	
Inconsistent	50.3	2.5 (1.7, 3.8)	58.1	2.6 (1.7, 4.0)	48.6	1.9 (1.3, 2.8)
Never	47.5	2.1 (1.4, 3.1)	56.7	2.4 (1.8, 3.3)	50.8	1.8 (1.3. 2.6)
Ever had oral sex^2^						
Yes	43.5	1.0 (0.7, 1.6)	43.8	1.0 (0.7, 1.4)	41.7	1.1 (0.7, 1.5)
No (reference)	41.7		46.4		41.2	
Self assessment of HIV risk						
No/low risk (reference)	24.7		25.2		26.8	
Moderate risk	53.4	3.6 (2.0, 6.4)	58.3	3.8 (2.6, 5.6)	60.0	3.0 (1.2, 7.7)
High risk	58.7	4.1 (3.0, 5.8)	61.5	3.9 (2.9, 5.3)	53.3	2.9 (2.1, 3.9)
Any chance that any current sex partner is HIV+						
Yes	51.0	3.2 (2.1, 4.9)	62.4	4.7 (2.9, 7.8)	51.1	2.6 (1.6, 4.3)
No (reference)	23.4		20.9		27.2	
Don't know	49.1	3.0 (2.0, 4.3)	48.2	3.1 (2.0, 4.6)	41.6	1.8 (1.2, 2.8)
Reported genital symptom at baseline						
Yes	55.9	1.9 (0.9, 4.0)	46.7	1.1 (0.4, 3.2)	70.4	4.2 (1.7, 10.2)
No (reference)	41.4		46.1		40.4	

1 Each row represents one bivariable model including age and the predictor of interest.

2 Anal sex, vaginal cleansing and vaginal drying were too infrequently reported to be assessed as a predictor of HIV prevalence (see Table 1).

**Table 3:** Determinants of Prevalent HIV Infection in the Cross-Sectional Studies – Multivariable Models.

Determinant	Ladysmith (N = 798)	Edendale (N = 1,084)	Pinetown (N = 891)
	Adjusted OR (95% CI)	Adjusted OR (95% CI)	Adjusted OR (95% CI)
Age (year)	1.12 (1.07, 1.17)	1.16 (1.12, 1.20)	1.13 (1.09, 1.18)
Highest level of education achieved:			
Some/completed primary education	1.87 (0.59, 5.94)	12.77 (2.93, 55.66)	4.57 (1.50, 13.95)
Some/completed high school	1.92 (1.09, 3.40)	6.72 (2.02, 22.40)	3.21 (1.21, 8.53)
Some/completed tertiary education (reference)			
Average monthly income			
0-R500 (reference)			
>R500	0.90 (0.59, 1.37)	1.46 (0.62, 3.41)	0.45 (0.26, 0.76)
Number of sex partners in last 3 months			
1 (reference)			
More than 1	1.95 (1.09, 3.50)	1.02 (0.58, 1.79)	1.27 (0.76, 2.12)
Condom use in last 7 days			
Always (reference)			
Inconsistent	1.89 (1.16, 3.07)	2.27 (1.39, 3.72)	1.24 (0.71, 2.16)
Never	1.27 (0.78, 2.06)	1.40 (0.96, 2.03)	1.07 (0.65, 1.76)
Self assessment of HIV risk			
No/low risk (reference)			
Moderate risk	2.72 (1.35, 5.46)	3.54 (2.29, 5.47)	2.29 (0.65, 8.04)
High risk	3.16 (2.10, 4.75)	3.15 (2.12, 4.67)	2.12 (1.32, 3.40)
Any chance that any current sex partner is HIV+			
Yes	2.61 (1.55, 4.40)	2.90 (1.62, 5.21)	2.58 (1.33, 5.03)
Don't know	2.36 (1.48, 3.78)	2.34 (1.46, 3.76)	1.56 (0.89, 2.72)
No (reference)			

### HIV incidence

**Figure 1:**
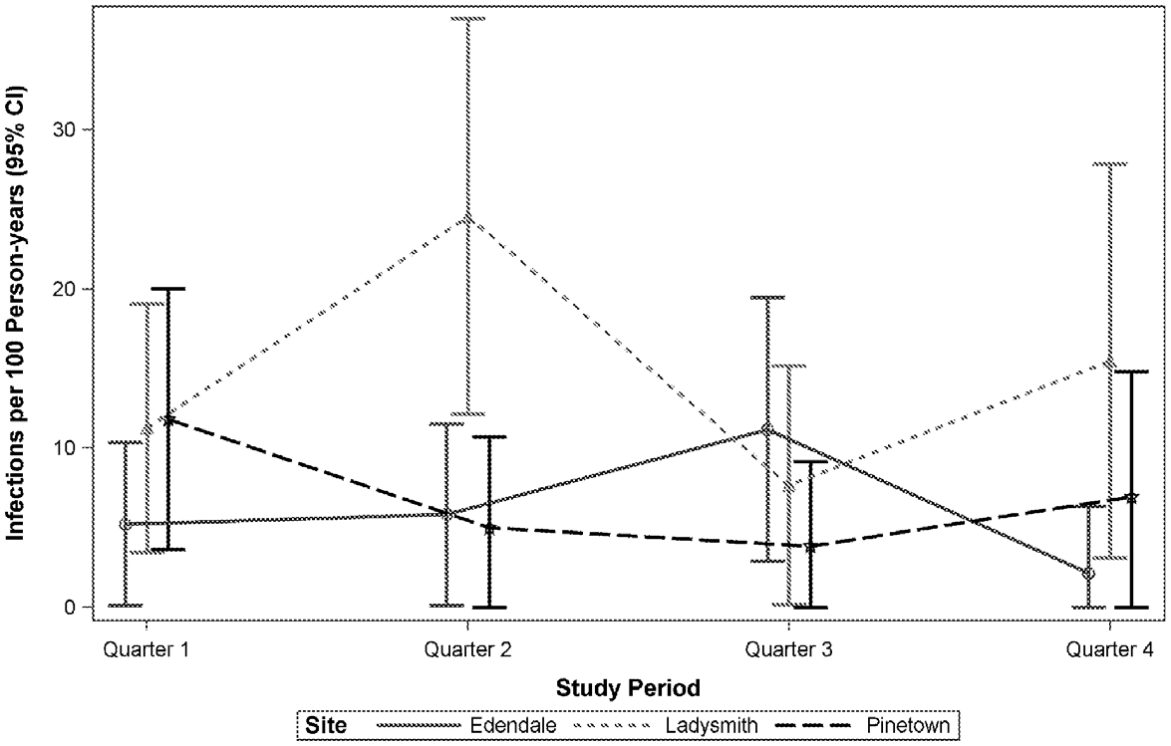
HIV incidence in the prospective cohort studies. Women enrolled in the 12-month cohort studies visited the CRC at 3, 6, 9, and 12 months after enrollment for HIV testing. HIV incidence rates were calculated based on a Poisson distribution with PY at risk in the denominator. They are expressed as number of cases per 100 PY with 95% confidence intervals. HIV infection was assumed to have occurred at the mid-point between the last available negative test and first positive test.

Overall HIV incidence rates based on seroconversions during the 12-month follow-up period in the cohort studies were 14.8/100 PY (95% CI 9.7, 19.8) in Ladysmith, 6.3/100 PY (95% CI 3.2, 9.4) in Edendale, and 7.2/100 PY (95% CI 3.7, 10.7) in Pinetown (Table 3). No clear trends in incidence rates over time could be discerned (Figure 1). Statistically significant predictors of HIV seroconversion were not identified, most likely due to limited statistical power, with the following exceptions: reporting 3 or more sex partners in the last 3 months (compared to 1 or 2 sex partners), and reporting genital symptoms at baseline, were associated with HIV seroconversion in Edendale (data not shown). The adjusted HIV incidence rates estimated by cross-sectional BED testing are shown in Table 4.

**Table 4:** HIV and Pregnancy Incidence Rates in the Prospective Cohort Studies.

	Ladysmith	Edendale	Pinetown
HIV incidence after 12 months	14.8 (9.7, 19.8)	6.3 (3.2, 9.4)	7.2 (3.7, 10.7)
HIV incidence in first 6 months	17.4 (10.3, 24.5)	5.5 (1.7, 9.3)	8.6 (3.5, 13.7)
HIV incidence in second 6 months	11.0 (4.2, 17.8)	7.3 (2.3, 12.4)	5.2 (0.6, 9.8)
HIV incidence BED adjusted (155 days; 1.7%) [17]	15.0 (10.1, 19.9)	10.2 (6.8, 13.7)	11.6 (7.6, 15.7)
HIV incidence BED adjusted (187 days; 1.7%) [17], [18]	12.5 (8.4, 16.6)	8.5 (5.6, 11.4)	9.7 (6.3, 13.0)
Pregnancy incidence after 12 months	5.7 (2.6, 8.7)	3.1 (0.9, 5.2)	6.3 (3.0, 9.6)
Pregnancy incidence in first 6 months	7.4 (2.8, 12.0)	2.0 (0, 4.4)	7.0 (2.4, 11.6)
Pregnancy incidence in second 6 months	3.2 (0, 6.7)	4.4 (0.5, 8.3)	5.3 (0.7, 9.9)

### Pregnancy prevalence and incidence

**Figure 2:**
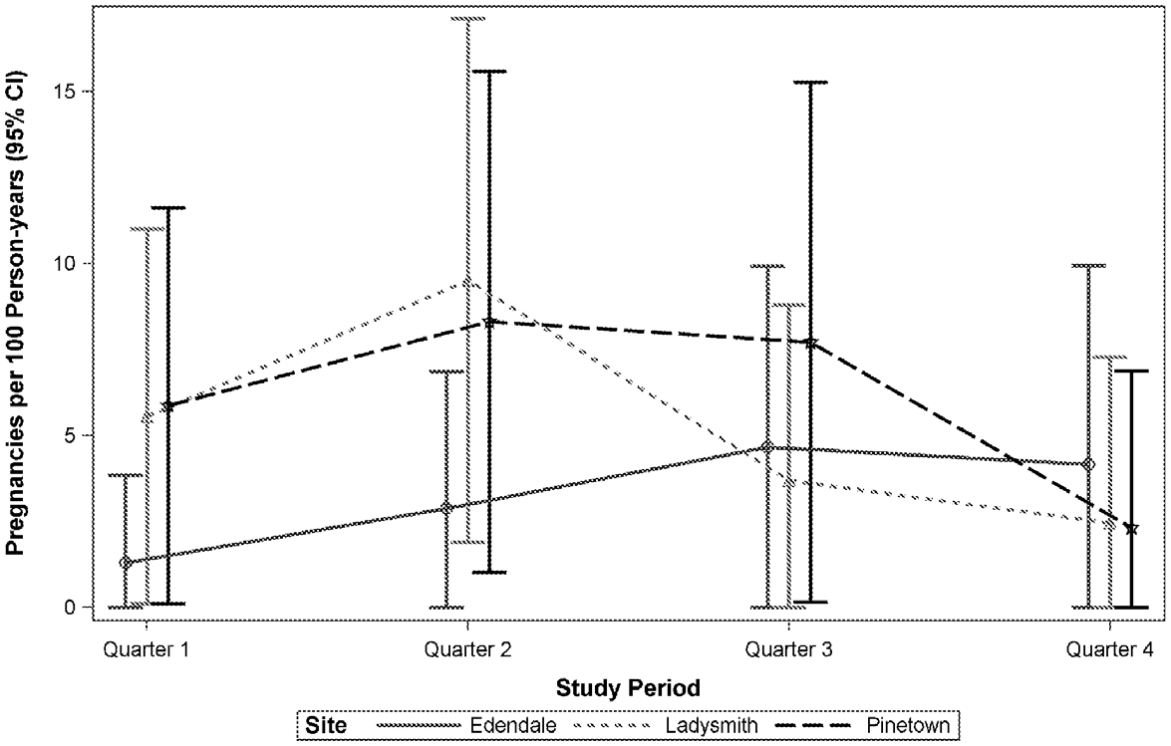
Pregnancy incidence in the prospective cohort studies. Urine pregnancy tests were done at each study visit (screening, enrollment, and 3, 6, 9, and 12 months after enrollment in the cohort study). If test result was positive, the participant was to continue on study for follow-up per protocol. Estimated date of conception and estimated due date were to be recorded. If possible, follow-up was to continue for pregnancy outcome. Contraceptive counseling was provided and condoms were dispensed at each study visit.

The pregnancy prevalence rates in the cross-sectional studies were low at all three CRCs in accordance with the recruitment strategy (only women reporting not to be pregnant were eligible for study participation): 2.6% (95% CI 1.6, 4.0) in Ladysmith, 4.1% (95% CI 3.0, 5.4) in Edendale, and 1.5% (95% CI 0.8, 2.5) in Pinetown. Pregnancy was associated with inconsistent condom use (age-adjusted OR 3.5, 95% CI 1.1, 11.4) and self-reported genital symptoms (age-adjusted OR 4.2, 95% CI 1.2, 15.2) in Ladysmith, and with ‘never used condoms’ (age-adjusted OR 4.1 (95% CI 1.8, 9.7) and self-reported moderate or high HIV risk (age-adjusted OR 3.7 (95% CI 1.5, 9.6) and 3.8 (95% CI 1.7, 8.8), respectively) in Edendale. In the cohort studies, overall pregnancy incidence for the 12-month period was 5.7 (95% CI 2.6, 8.7) in Ladysmith, 3.1 (95% CI 0.9, 5.2) in Edendale, and 6.3 (95% CI 3.0, 9.6) in Pinetown. Again, no trends were observed over time (Figure 2).

## Discussion

Our data confirm that HIV prevalence and incidence continue to be high in sexually active women aged 18–35 years living in peri-urban areas of KwaZulu-Natal. Our prevalence rates are similar to those reported in the 2009 and 2010 national antenatal surveys but higher than those reported in the 2008 HSRC population-based household survey (26% for women and men combined and for all districts of KwaZulu-Natal combined) [3]–[5]. The latter is most likely due to the fact that the HIV prevalence is higher in South African women than in men; unfortunately, only aggregate data were reported [5]. Compared to women aged 20–34 participating in the HSRC survey, a higher proportion of our study participants reported having had 2 or more partners in the last 3 months (8–13% versus 4%) and a slightly lower proportion reported condom use during the last sex act (47–62% versus 67%) [5].

Our HIV incidence rates of 6.3 to 14.8 per 100 PY suggest that HIV transmission is still rampant in KwaZulu-Natal. Our incidence rates for Edendale (6.3/100 PY; uMgungundlovu district) and Pinetown (7.2/100 PY; eThekwini district) fall within the range of rates recently reported for urban and rural women in eThekwini and uMkhanyakude districts (6.4/100 PY among urban women and 6.5/100 PY among rural women aged 14–30 years [7] and 3.6/100 PY among rural women aged 15–55 [10]). Our incidence rate for Ladysmith (14.8/100 PY; uThukela district), however, was substantially higher than any of these rates and we were not able to identify any reported incidence rates for Ladysmith or uThukela district to compare ours to. The higher incidence rate in Ladysmith cannot be explained by a higher HIV prevalence. HIV prevalence in uThukela district was slightly higher than in the other districts in 2009 but fell with almost 10% to a relatively low level of 37% in 2010 [3], [4]. We also found hardly any significant differences in demographics (age, education and marital status) and sexual behavior between the three study populations. A higher proportion of women in Ladysmith had an average monthly income higher than R500 than in the other two study areas (32% versus 4 and 13%) but income was not associated with HIV seroconversion. Data on male circumcision, alcohol use, the presence of laboratory-confirmed sexually transmitted infections, and migration were not collected; temporary migration to other urban areas (such as Johannesburg and Durban) for work may fuel the HIV epidemic in Ladysmith more than in the other districts. Furthermore, HIV incidence was particularly high in the second quarter of the study, which is when recruitment in more remote rural areas of uThukela district was initiated. This population had not previously had access to high quality HIV counseling and testing services.

As expected, HIV incidence rates based on the adjusted BED-CEIA results were higher than those based on seroconversions per 100 PY for the two sites with lower HIV incidence (Edendale and Pinetown; Table 4) [17], [18]. However, the confidence intervals overlap substantially for all three study sites.

While HIV incidence at the three study sites seems sufficiently high for implementation of HIV microbicide efficacy trials, retention rates would have to be improved (currently 43–70%) and pregnancy incidence would have to be reduced. Women in our studies were required to use a condom and a hormonal method of contraception but the high pregnancy incidence rates indicate that these methods were not used correctly and consistently.

A few limitations of our data should be noted. The eligibility criteria for study participation may have limited generalizability of our results. The HIV prevalence rates apply only to young, sexually active women who were not known to be HIV-infected or pregnant, and who agreed to be tested regularly for HIV. The total number of seroconversions in each prospective cohort study were low (16–33 cases) and the 95% confidence intervals were therefore wide. The low retention rates of our cohort studies (43–70%) may have biased our HIV incidence estimates based on seroconversions. We do not have any indications that the women who left the cohort studies early were at higher or lower risk of HIV acquisition than the women who remained in the study but we cannot be certain. The 95% confidence intervals of the cross-sectional BED-based HIV incidence estimates were also wide. Furthermore, we did not measure local false-recent rates or window periods and could therefore not adjust our BED estimates as recommended by WHO [19].

In conclusion, HIV prevalence and incidence remain very high in sexually active women living in peri-urban areas of KwaZulu-Natal. HIV prevention interventions in these populations should be strengthened.
